# Umbilical artery thrombosis: Two case reports

**DOI:** 10.1097/MD.0000000000018170

**Published:** 2019-11-27

**Authors:** Huanxi Li, Qufeng Wu, Wei Wei, Xueyan Lin, Xueqin Zhang

**Affiliations:** Department of Obstetrics, Women and Children's Hospital, School of Medicine, Xiamen university, Xiamen, China.

**Keywords:** pregnancy, single umbilical artery, umbilical artery thrombosis

## Abstract

**Rationale:**

The umbilical cord is the way to exchange gas, supply nutrients, excrete metabolized. Thrombosis of the umbilical cord leads to fetal hypoxia, which jeopardizes fetal health and can cause fetal death. Umbilical vessel thrombosis, which is rarely reported, is difficult to detect prenatally.

**Patient concerns:**

Both pregnant women had an unremarkable pregnancy course until a routine ultrasound scan in the third trimester showed a single umbilical artery. However, one umbilical vein and 2 umbilical arteries were seen during an ultrasound examination at 32 weeks. Case 2 had a better pregnancy outcome because of the timely discovery of this complication.

**Diagnosis:**

Both cases were diagnosed as umbilical artery thrombosis.

**Interventions:**

The first patient received no interventions until they reported decreased fetal movements and gradually disappear. The second patient underwent an emergency cesarean section.

**Outcomes:**

In Case 1, an emergency ultrasound examination showed intrauterine fetal death, and the patient vaginally delivered a stillborn child weighing 3300 g in a day. In Case 2, a female neonate weighing 2860 g was delivered by cesarean section, and exhibited Apgar scores of 10 and 10 at 1 and 5 minutes.

**Conclusion:**

In the late-term abortions, obstetricians should be vigilant if ultrasound imaging shows suspected umbilical vascular thrombosis or shows 1 umbilical artery when there had previously been 2. The fetus should be closely monitored and interventions implemented as early as possible to improve the prenatal detection rate of umbilical vessel thrombosis and avoid adverse pregnancy outcomes.

## 1 Introduction

The umbilical cord comprises one umbilical vein and two umbilical arteries protected by Wharton jelly. Umbilical cord thrombosis is associated with increased fetal and perinatal morbidity and mortality. Umbilical artery thrombosis (UAT) is a rare pregnancy complication, with an estimated incidence ranging from 0.0025% to 0.045% of gestations.^[1]^ UAT is difficult to detect prenatally, so the diagnosis and clinical management of this complication remain a challenge. Herein, we report 2 cases of UAT which were detected prenatally via ultrasound examination.

## 2 Case report

### 2.1 Case 1

**Figure 1:**
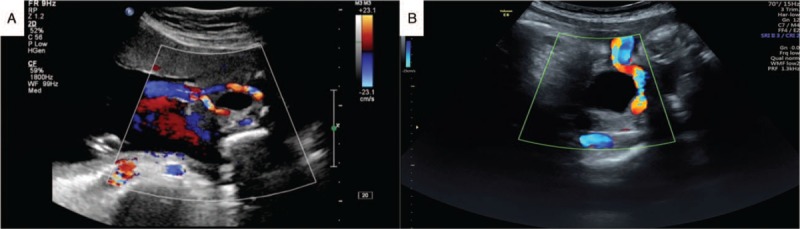
Case 1 of umbilical artery thrombosis. (A) The routine ultrasound scan showed 1 umbilical vein and 2 umbilical arteries. (B) Single umbilical artery blood flow imaged using Doppler ultrasound at the level of the bladder.

A 26-year-old, primiparous, pregnant woman had an unremarkable pregnancy course until 37 weeks. The routine first and the second-trimester ultrasound scans exhibited normal findings. One umbilical vein and two umbilical arteries were observed during an ultrasound examination at 32 weeks (Fig. 1A). At 36 weeks and 5 days of gestation, a routine ultrasound scan showed a single umbilical artery, and the flow parameter of umbilical artery and middle cerebral artery for the fetus was well into the normal range (Fig. 1B). Non-stress test of fetal heart rate monitoring was responsive. Two days later, at 37 weeks, the pregnant woman reported decreased fetal movements and gradually disappear. An emergency ultrasound examination showed intrauterine fetal death. The patient was admitted to hospital and was immediately induced with 0.5% oxytocin. The patient vaginally delivered a stillborn child weighing 3,300 g in a day. There were no obvious abnormalities in the appearance of the stillborn child. Obvious congestion was noted in the umbilical cord near the umbilical round meaning a thrombosis of blood vessels. No chromosomal aneuploidies were detected, and the fetal necropsy showed no abnormalities. With respect to the pathological examination of the umbilical cord and placenta, three umbilical vessels, consistent with UAT, with complete occlusion of blood vessels.

### 2.2 Case 2

**Figure 2:**
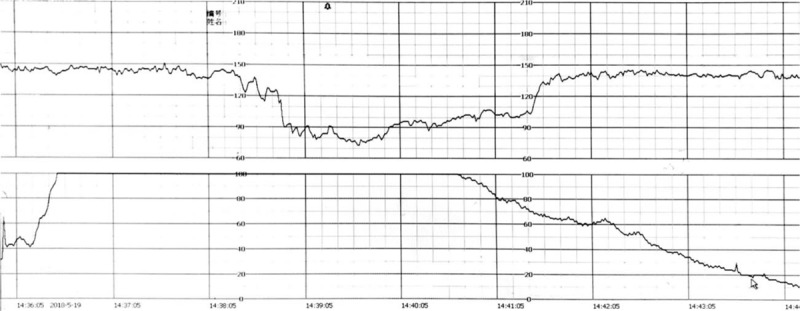
Case 2 of umbilical artery thrombosis. Fetal heart rate monitoring showed extended deceleration.

**Figure 3:**
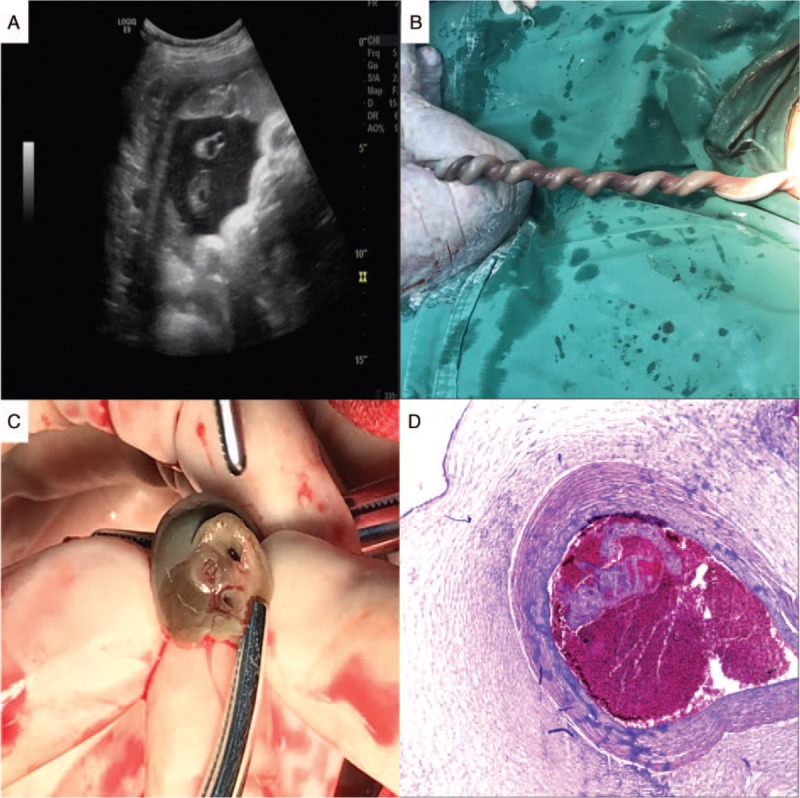
Case 2 of umbilical artery thrombosis. (A) In the ultrasound examination, one of the umbilical arteries was poor in transparent sound and was hyperechoic. (B) Gross imaging of the umbilical cord, demonstrating its highly coiled appearance. (C) Gross appearance of the umbilical cord, demonstrating the presence of thrombosis in the umbilical vessel. (D) Upon microscopic examination, a thrombosis was found in the umbilical artery on the chorionic plate.

A 25-year-old old, multiparous, pregnant woman had an unremarkable pregnancy course until 38 weeks. Routine ultrasound scans at 24 weeks, 32 weeks, and 36 weeks exhibited normal findings. One umbilical vein and 2 umbilical arteries were observed during an ultrasound examination at 36 weeks. At all antenatal visits, auscultation of the fetal heart was unremarkable. At 38 weeks of gestation, out-patient fetal heart monitoring showed severe, prolonged fetal bradycardia, in which the fetal heart rate remained between 70 and 100 beats per minute for more than 2 minutes (Fig. 2). The patient was admitted to hospital, and an emergency ultrasound examination showed a single umbilical artery. Given the results of the previous ultrasound examination, UAT was suspected (Fig. 3A). After informed consent was obtained from the patient and her family, we performed an emergency cesarean section. A female neonate weighing 2860 g was delivered, and exhibited Apgar scores of 10 and 10 at 1 and 5 minutes, respectively. During the operation, the umbilical cord was extremely torsional in 20 laps (Fig. 3B and C). One of the umbilical arteries show yellow dye and blockage of blood flow. With respect to the pathological examination of the umbilical cord and placenta, three umbilical vessels, umbilical cord excessively rotating with a recent UAT (Fig. 3D).

## 3 Discussion

### 3.1 Etiology of umbilical vascular thrombosis

Umbilical vascular thrombosis is a rare pregnancy complication. Umbilical vein thrombosis is more common than UAT, but UAT is associated with increased fetal and perinatal morbidity and mortality. Venous, venous and arterial, and arterial thrombosis have been reported to occur in 70%, 20%, and 10% of cases, respectively.^[2]^ Its pathogenesis and mechanism have not yet been identified. According to Heifetz, umbilical vascular thrombosis occurs in around 0.08% of deliveries, 0.1% of postpartum autopsies, and 0.4% of high-risk pregnancies.^[3]^

Thrombosis can be formed in the circulation of umbilical vein or artery. It can accumulate in capillary blood vessels, villus capillaries, trunk vessels, and umbilical vessels. This may be caused by mechanical injury or abnormal anatomy of the umbilical cord, for example twining, twisting, tieing, excessive helix, mecism, compression, and abnormal attachment.^[4,5]^ Cord anomalies may induce flow stasis and thrombosis of the umbilical artery. In Case 1, no umbilical cord torsion was noted, though this does not rule out the existence of a small twist resulting in the umbilical cord not being fully protected by Wharton jelly. It was easy to be pressed and caused umbilical vascular thrombosis. In Case 2, the umbilical cord, particularly the central part of the cord, was extremely twisted. Umbilical vascular thrombosis may also be related to abnormal fetal coagulation function, abnormal maternal plasma glucose profiles, infection, and smoking. Umbilical vascular thrombosis has also been associated with thrombotic disease, vascular endothelial injury, increased platelet number and activity, increased blood coagulation, decreased anticoagulant and fibrinolytic activity, and hemodynamic abnormalities.^[6,7]^

### 3.2 The ultrasound scan in UTA

UAT is associated with increased neonatal morbidity and mortality - Sato^[2]^ reported that of seven cases of UTA, 2 infants were stillborn, one had a neonatal stroke, 1 was born with congenital anomalies and died shortly after birth, and 1 had a complicated neonatal course with prolonged admission to the neonatal intensive care unit. None of the infants had a normal course. Heifetz^[3]^ showed that 80% of cases of stillbirth caused by umbilical vascular thrombosis were due to UAT. Shilling et al^[8]^ and Tanaka et al^[9]^ believe that there is a significant relationship between UAT and fetal intrauterine growth restriction.

Over recent decades, prenatal care has significantly improved, and the evolution of ultrasound imaging has been key in this advancement. However, the prenatal diagnosis of UAT remains a clinical challenge. In our cases, UAT was diagnosed prenatally via ultrasound examination. Umbilical vein thrombosis is more common, and reports of cases prenatally diagnosed as UAT by ultrasound examination are limited.^[10,11]^ A transverse 2-dimension ultrasound scan of the umbilical cord shows 3 rings - 1 big ring, the umbilical vein, and 2 small rings, the umbilical arteries. When one of the umbilical arteries is embolized which show as arterial blood flow blocked, single umbilical artery of cord is easily misdiagnosed by ultrasound examination. One way of avoiding this situation need abundant clinical experience for ultrasound doctors and to compare before-and-after ultrasound imaging of the umbilical arteries and umbilical vein. Klaritsch et al^[12]^ reported the characteristic ultrasound finding of UAT, the occluded artery in parallel with the remaining artery and surrounded by the uterine vein, which resembled ‘an orange grabbed by a hand’. In an ultrasound image, a highly curved ‘C-shaped’ vein surrounding the arteries may represent hypercoiling of the umbilical cord. This is a reproducible and novel sign of UAT. In Case 2, in the ultrasound examination, one of the umbilical arteries was poor in transparent sound and was hyperechoic. In cases like this ultrasound examination, we ultrasound examination suspect UAT Combining with the past ultrasound examination of the same patient. Although the number of umbilical arteries can be confirmed by blood flow evaluation by color Doppler at the level of the fetal bladder, in cases of single umbilical artery, it is difficult to distinguish between primary agenesis and secondary obstruction. The aforementioned finding of ‘an orange grabbed by a hand’ is completely different to the cross section of primary agenesis, and thus can successfully differentiate between them.

## 4 Conclusion

Umbilical vascular thrombosis is a rare but serious pregnancy complication. In the late-term abortions, obstetricians should be vigilant if ultrasound imaging shows suspected umbilical vascular thrombosis or shows 1 umbilical artery when there had previously been 2. The fetus should be closely monitored and interventions implemented as early as possible to improve the prenatal detection rate of umbilical vessel thrombosis and avoid adverse pregnancy outcomes.

## Author contributions

**Conceptualization:** Huanxi Li, Qufeng Wu.

**Data curation:** Qufeng Wu.

**Formal analysis:** Qufeng Wu.

**Funding acquisition:** Qufeng Wu.

**Investigation:** Xueyan Lin.

**Methodology:** Xueyan Lin.

**Project administration:** Xueyan Lin.

**Resources:** Wei Wei.

**Supervision:** Wei Wei.

**Validation:** Wei Wei.

**Visualization:** Wei Wei.

**Writing – original draft:** Huanxi Li.

**Writing – review & editing:** Xueqin Zhang.
