# Umbilical artery thrombosis: Two case reports: Erratum

**DOI:** 10.1097/MD.0000000000018563

**Published:** 2020-01-10

In the article, “Umbilical artery thrombosis: Two case reports”,^[1]^ which appears in Volume 98, Issue 48 of *Medicine*, Quanfeng Wu's name appeared incorrectly as Qufeng Wu.
